# CA^2^PNet: a context-aware multi-scale architecture with adaptive attention and progressive dilated convolutions for biomedical image segmentation

**DOI:** 10.3389/frai.2026.1802033

**Published:** 2026-06-05

**Authors:** Aman Kumar Singh, Ashish Ranjan, Manas Ranjan Prusty, Nitish Katal

**Affiliations:** 1 School of Computer Science and Engineering, Vellore Institute of Technology, Chennai, India; 2 Centre for Cyber Physical Systems, Vellore Institute of Technology, Chennai, India

**Keywords:** attention mechanisms, breast tumor segmentation, context-aware feature extraction, dilated convolutions, medical image segmentation, multi-scale representation learning, polyp segmentation

## Abstract

**Background and objectives:**

Accurate medical image segmentation remains a challenging task in computer-aided diagnosis because of the intricacies and the variability in the biomedical data in terms of the anatomical complexity, inter-patient diversity, class imbalance, and irregular morphological patterns.

**Methods:**

In the present work, a Context Aware Adaptive Progressive Network (CA^2^PNet) is proposed. The foundational architecture of CA^2^PNet is inspired from DeepLabV3+ and FusionNet and introduces four key modifications by the incorporation of (a) the spatial attention module (SAM) to emphasize discriminative spatial regions, (b) Global Max Pooling to strengthen contextual representation and suppress background noise, (c) an enhanced Spatial Pyramid Pooling for robust multi-scale feature extraction, and (d) progressive dilated convolutions to expand the receptive field while preserving fine structural details. The incorporation of these modules enables the simultaneous refinement of the extraction of local features as well as preserving the global context.

**Results:**

CA^2^PNet offered a mean intersection of union for 85.15 and 82.78% for the Kvasir-SEG and BUSI datasets, respectively, surpassing state-of-the-art models. The statistical tests also validate the robustness of the proposed model.

**Conclusions:**

The proposed work demonstrates that the embedding of multi-scale features at each encoder stage along with decoupled decoding aids in overcoming the limitation of resolution loss in classical segmentation architectures; thus, resulting in superior boundary adherence and scale-invariant segmentation.

## 1 Introduction

In computer-aided medical diagnosis, image segmentation has become one of the indispensable components for various downstream clinical tasks like tracking the disease progression, surgery planning, organ boundary demarcation, and tumor quantification. The clinical ecosystem relies heavily on automated segmentation across imaging modalities, such as magnetic resonance imaging (MRI), computed tomography (CT), endoscopy, digital pathology, ultrasound, etc., to manage the scale, complexity, and diagnostic precision in routine workflows. Traditionally, systems were based on manual feature engineering, rule-based methods and classical machine learning, which significantly rely on filters, edge detectors, texture descriptors, and morphological operations. These systems were highly sensitive to noise, illumination, and anatomical heterogeneity, and thus, they were not able to assure diagnostic performance across patient populations, imaging devices, etc., limiting their generalizability and clinical trustworthiness (Yu et al., 2023). On the other hand, deep learning has revolutionized the field of medical diagnosis, wherein these deep learning models automatically learn the textual and/or anatomical features and their contextual dependencies from the data directly; and the use of advanced mechanisms like attention modules and feature fusion strategies further enhances the model's ability to learn ambiguous boundaries, low-contrast regions, and modality-specific artifacts. This shift from handcrafted features to driven feature extraction has significantly improved robustness, accuracy, and adaptability and has enabled such diagnosis systems to perform reliably across diverse imaging conditions and clinical scenarios.

Despite these advancements, challenges still exist in medical image segmentation. The traditional computer-aided design (CAD)-based segmentation models are reliant on thresholding, active contours, watershed algorithms, etc. and often fail to generalize well in real-world settings, leading to inaccurate boundary localization because of the challenges posed by the variation in tissue appearance, low contrast between the pathological and nominal regions, irregular shape of the lesion, and presence of noise or artifacts (Pham et al., 2000; Yu et al., 2023). Along with that, the modality-specific issues like speckle noise in ultrasound (Avola et al., 2022), motion artifacts in endoscopy (Ali et al., 2019), and partial volume effects in MRI (Tohka, 2014; Zaitsev et al., 2015), further aggravate the challenges. To address these, the deep-learning-based models have transformed the medical imaging segmentation by enabling direct learning from the data in a hierarchical manner to obtain robust feature representations. These models primarily feature encoder–decoder architectures, and the use of multi-scale feature extraction and attention mechanisms further allows these models to extract fine-grained structured details, thus enabling robust segmentation (Wang et al., 2022).

Therefore, for reliable segmentation, the need for robust feature extraction becomes critical: the diagnostic ability of the model is fundamentally dependent on the quality and the discriminative power of the captured features, and without a strong feature representation that can isolate the pathological patterns from the surrounding tissues, the models will struggle to maintain consistency across diverse cases (Jiao et al., 2025). In particular, the polyps in colonoscopy and lesions in breast ultrasounds exhibit highly ambiguous boundaries and irregular morphologies; this necessitates robust feature extractors capable of capturing both fine-scale textures and broader contextual relationships (Elizar et al., 2022). Thus, one of the critical problems with classical convolutional neural networks (CNNs) is that they extract features using kernels of fixed sizes (e.g., 3 × 3), whereas the pooling layers increase the receptive field, but this comes at the cost of the resolution loss, and deep networks with aggressive downsampling often “pool away” these tiny features.

In the case of the encoder–decoder architectures, the primary cause for this is the difference between the information available at the different stages of the deep network. The features at the shallow layers or the early stages of the encoder offer higher spatial fidelity but low semantic meaning, whereas the deeper layers or decoder offer high semantic information but low spatial fidelity. The shallow layer detects the edges, simple textures etc., but the deeper layer has information that a tumor exists but has lost all the information of its precise boundary because of repeated downsampling. Thus, one of the fundamental challenges in semantic segmentation is to enable the robust transfer of spatial information from the encoder to the decoder without introducing noise. A naïve concatenation between the encoder–decoder stages will dump high-frequency noise into the decision-making layers of the decoder, thus confusing the model (Li M. et al., 2025). This inability to extract robust features can lead to adverse clinical outcomes, wherein the diagnosis will be uncertain (Teng et al., 2022), will reduce the efficacy and efficiency of the clinical workflows (Wang H. et al., 2025), and will result in missed early detections and treatment planning (Diwakar et al., 2023).

To address the scale dilemma and the loss of spatial details, a diverse array of multi-scale extraction techniques has been proposed in the literature, wherein they aim to present a pyramidal view of the data, allowing for the simultaneous processing of both the fine details and the global context. The feature pyramid structures have been effectively employed in medical imaging. These methods include the following: feature pyramid networks (FPN) to ensure strong multi-scale feature maps at each level; dilated convolution and Atrous spatial pyramid pooling (ASPP), which aim to expand the receptive field without the pooling associated resolution loss; asynchronous convolutions, which feature a cross-shaped receptive field rather than a square one; multi-scale residual blocks (MSRB) ensuring that the network considers features of varying granularity even at a single depth; deep supervision, which attaches auxiliary classification heads to the intermediate layers of the decoder and the loss is calculated at multiple scales (e.g., 1/8, 1/4, 1/2 resolution) and summed, thus forcing the intermediate layers to produce semantically meaningful features, ensuring that the multi-scale representations are discriminative.

Apart from the extraction of a rich palette of multi-scale features, fusion strategies also play a pivotal role; for example, in classical UNet, the skip connections concatenate the encoder features to the decoder. However, this naïve concatenation introduces noise in the decoding stages. To address this in UNet architecture, Zhou et al. introduced dense and nested skips to propose UNet++. Another strategy to address noise propagation is to employ attention-gated fusion, wherein the decoder features are used as gating signals to highlight the regions of interest and suppress background noise. Some researchers have also explored bi-directional FPNs, which feature both top-down and bottom-up pathways and introduce learnable weights at the fusion nodes. Some of the recent studies have employed graph neural networks to model the non-Euclidean relationships between features and enable addressing the view-specific noise. Recently, transformer-based segmentation models like TransUNet and Swin-UNet have been introduced to model the long-range dependencies. However, the self-attention in transformers scales quadratically with sequence length, and recently, to address this bottleneck, architectures like Mamba (HCMUNet and Mamba-UNet++) have introduced the state space models with linear complexity. To address these challenges, we have proposed a Context Aware Adaptive Progressive Network (CA^2^PNet), wherein the key contributions of the proposed work are listed as follows:

- CA^2^PNet introduces four key modifications by the incorporation of (a) spatial attention module (SAM) to emphasize discriminative spatial regions, (b) Global Max Pooling (GMP) to strengthen contextual representation and suppress background noise, (c) an enhanced Spatial Pyramid Pooling (SPP) for robust multi-scale feature extraction, and (d) progressive dilated convolutions to expand the receptive field while preserving fine structural details.
- The encoder features an Enhanced Multi-Scale Feature Extractor, which integrates parallel multi-scale context blocks at each state. The module enables a fixed spatial resolution of $\frac{H}{4}\times \frac{W}{4}$, throughout the encoding process.
- Two-stage decoder network, wherein both the first stage fuses all the features from all encoder stages to locate the lesion; whereas the second stage integrates the attention-refined high-resolution features *via* a SAM to precisely recover the object boundaries.
- The work has been evaluated for the segmentation of the lesions in ultrasound images for breast cancer diagnosis and also for the segmentation of the gastrointestinal polyps using endoscopic images; and a mean intersection-over-union of 0.8278 and 0.8515 is achieved on evaluation on BUSI and the Kvasir-SEG datasets, respectively.
- Statistical validation using a non-parametric Mann-Whitney U test and a Paired *T*-Test on the 5-fold cross-validation results has also been incorporated to validate the robustness of the model.

## 2 Existing works

Qualitative analysis of medical images has played a pivotal role in precision medicine, and image segmentation is one of the foundational tasks in computer-based diagnosis. There has been an upsurge in the architectural diversity of deep learning-based methods in recent years, wherein these segmentation architectures have evolved from classical UNet to transformer-based architectures. These methods are being widely used in the segmentation of cancerous lesions, and the need for accurate segmentation is critical given the substantial global burden of cancer. Colorectal cancer remains the second leading cause of cancer mortality globally, and it usually starts from adenomatous polyps in the colon. The screening is done using colonoscopy; however, the polyp miss rate can vary between 12 and 30% wherein the sessile polyps might be prone to oversight. Conversely, breast cancer is the most prevalent malignancy in women, and ultrasound is a critical modality for screening. However, the interpretation of the ultrasound is highly subjective to the operator and is plagued by artifacts like acoustic shadowing, posterior enhancement, and speckle noise. Kvasir-SEG and BUSI are benchmark datasets for these two modalities.

In Wang Z. et al. (2025), an encoder-decoder based architecture is proposed for the segmentation of gastrointestinal polyps, wherein a pre-trained ConvNeXt model is used in the encoder and residual transformer block in the decoder and the encoder and decoder are connected *via* a cross-attention mechanism. The study considers a Kvasir-SEG dataset wherein a dice coefficient of 0.8657 and a mean intersection over union (IOU) of 0.8021 is achieved along with balanced precision and recall of 0.9023 and 0.8786, respectively. The model exhibited superior performance when compared to CNN and transformer-based baselines; however, the performance reduces in the case of small and flat polyps, and the inclusion of a cross-attention mechanism increases the computational complexity. In Singh et al. (2025), a ResSegNet++ model is proposed, which features two parallel encoder-decoder networks with a squeeze and excitation block along with skip connections of the model. The integration of the squeeze and excitation block improves the representation power of the network. The model has been evaluated on Kvasir-SEG and CVC-ClinicDB datasets for polyp segmentation and an mIOU of 0.6680 and 0.6039 is reported for these datasets, respectively. DUCK-Net (Dumitru et al., 2023) features a novel encoder–decoder architecture for the segmentation of the polyps. It characterizes the DUCK module, which features dense connections, upscaling, convolution kernels, and knowledge distillation operations. The model has been evaluated on multiple datasets like Kvasir-SEG, CVC-ClinicDB, etc., and for the Kvasir-SEG dataset model, it achieves a dice score of 0.923. The DUCK-Net features a lightweight design and the use of knowledge distillation improve efficiency without sacrificing the accuracy; however, the performance drops for small/flat polyps. PolySEAG-Net (Liu D. et al., 2025) features an encoder-decoder segmentation architecture, wherein the feature learning is refined by integrating the squeeze-and-excite blocks with attention gates. The model achieves an IOU of 0.8025, a precision of 0.9238, and a recall rate of 0.8683 for the Kvasir-SEG dataset. In Song and Shin (2024), the use of generative models is considered to create synthetic polyp images and masks, wherein SemanticPolypGAN employs pseudo-length maps to layer polyps over colon surfaces, and RenderNet refines the masks; an mIOU of 0.8362 is obtained on the Kavsir-SEG dataset. In Zhang et al. (2024), VM-UNET-V2 is proposed, which integrates Mamba (Vision State Space) blocks and Semantic and Detail Infusion (SDI) modules within a UNet-like encoder–decoder structure offering linear complexity; and the model offers an mIOU of 0.8415 for the Kvasir-SEG dataset. ConvSegNet (Ige et al., 2023) proposes the integration of multi-scale feature fusion and attention mechanisms in the CNN-based segmentation architecture to handle polyps of varying sizes and refinement of the boundaries; on the Kvasir-SEG dataset, a dice score of 0.8618 is reported. In Tomar et al. (2021), a dual-decoder attention network (DDANet) is proposed for the segmentation of polyps, the model features dual decoder architecture wherein the first one performs segmentation and the second one act as an autoencoder to refine the feature representation using attention maps; and offers a mIOU of 78% on Kvasir-SEG dataset. In Zhang et al. (2020), an adaptive context selection (ACSNet) model is proposed for polyp segmentation, the architecture features local (LCA) and global (GCM) context modules with an adaptive selection module (ASM), which aggregates the local and global features *via* channel-wise attention. The architecture features a ResNet34 encoder with five blocks, paired with a decoder of equal depth. The skip connections are replaced by the LCA, GCM features are fed to the decoder, and the ASM integrates features adaptively. The model has been evaluated on the Kvasir-SEG dataset wherein a mIOU of 79.73% is achieved. In Fan et al. (2020), the PraNet model is proposed, featuring the Res2Net encoder, parallel partial decoder, and reverse attention modules for polyp segmentation, and offers a mIOU of 82.94% for the Kvasir-SEG dataset. In Lin et al. (2024), CswinDoubleU-Net is introduced, which features a double U-shaped network composed of CNNs and Swin Transformer, wherein the first U-shaped CNN extracts the local features while the second one extracts the global features; it has been evaluated for the Kvasir-SEG dataset, and a mIOU of 84.4% is reported. In Yeung et al. (2021), Focus U-Net is proposed, which features a focus-gate to combine the spatial and channel attention in a U-Net-like architecture and achieves an mIOU of 84.5% of the Kvasir-SEG architecture. In Huang et al. (2021), the HarDNet-MSEG is proposed, featuring a HarDNet68 backbone and cascaded partial decoder, evaluated on the Kvasir-SEG dataset, and it offers an mIOU of 84.8%.

In Li G. et al. (2025), a lightweight SMM-UNet is proposed, which includes a selective fusion mamba module and a multi-scale fusion mamba module and features a UNet-inspired encoder–decoder architecture. The SF-Mamba module ensures better delineation of the tumor boundary, whereas the MF-Mamba ensures the handling of lesions of various sizes. The work has been evaluated on the BUSI dataset, and an IOU of 0.688 is achieved. FET-UNet (Zhang H. et al., 2025) features a UNet-inspired architecture, wherein each encoder block features parallel ResNet34 and Swin Transformer branches with an advanced feature aggregation module (AFAM), wherein the AFAM features spatial self-attention and channel recalibration. The model offers an IOU of 0.747 for the BUSI dataset. In Anari et al. (2025), a UNet-inspired architecture is featured wherein the first stage of the encoder features three parallel branches of ResNet, DenseNet, and EfficientNet, and the decoder features a convolutional block attention module (CBAM) and non-local attention modules; the model has been evaluated on the BUSI dataset, wherein an IOU of 0.5305 is reported. In Aumente-Maestro et al. (2025), a multi-task framework is proposed featuring a prediction-refining module and deterministic oversampling to handle class imbalance; for the BUSI dataset, a DSC of 0.751 is reported. EMGA-Net (Huang et al., 2025) features an encoder–decoder architecture with attention-driven fusion, wherein it combines multi-scale group-mix (MGM) attention and edge feature enhancement (EFE) modules; the MGM modules aggregate the sparse global and local features for robust representation, and the EME block enables the enhancement of boundary precision. EGMA-Net offers a mIOU of 0.8137 for the BUSI dataset. In Zhang Y. et al. (2025), an encoder–decoder architecture is proposed wherein the encoder features multi-scale convolution blocks with the attention modules, and the decoder features edge refinement layers; the work has been evaluated on the BUSI dataset wherein an IOU of 0.7814 is attained. In Liu H. et al. (2025), a U-Net-inspired architecture is proposed featuring residual connections in the encoder, spatial hybrid convolution modules for extracting global features, channel attention mechanism, and multi-convolutional self-attention modules. In Jiang et al. (2023), a U-Net-based model with CNN-transformer hybrid encoder, coordinate residual block, enhanced channel self-attention transformer, and comprehensive dual attention module and cascaded decoder is proposed for the segmentation of lesions in breast ultrasound images.

The comparison has been summarized in Table 1 as follows:

**Table 1:** Summary of existing works.

References	Domain	Architecture and key components	Dataset	Performance metrics
Wang Z. et al. (2025)	Polyp	Enc-Dec: ConvNeXt (Enc) + Residual Transformer (Dec) with Cross-Attention.	Kvasir-SEG	Dice: 0.8657 mIOU: 0.8021
ResSegNet++ (Singh et al., 2025)	Polyp	Parallel Enc-Dec: Squeeze & Excitation (SE) blocks, skip connections.	Kvasir-SEG, CVC-ClinicDB	mIOU: 0.6680 (Kvasir) mIOU: 0.6039 (CVC)
DUCK-Net (Dumitru et al., 2023)	Polyp	DUCK Module: Dense connections, upscaling, knowledge distillation.	Kvasir-SEG	Dice: 0.923
PolySEAG-Net (Liu D. et al., 2025)	Polyp	Enc-Dec: SE blocks integrated with Attention Gates.	Kvasir-SEG	IOU: 0.8025 Prec: 0.9238
Song and Shin (2024)	Polyp	Generative: SemanticPolypGAN (pseudo-depth) & RenderNet (mask refinement).	Kvasir-SEG	mIOU: 0.8362
VM-UNET-V2 (Zhang et al., 2024)	Polyp	Mamba: Vision State Space blocks + SDI modules in UNet-like structure.	Kvasir-SEG	mIOU: 0.8415
ConvSegNet (Ige et al., 2023)	Polyp	CNN-based: Multi-scale feature fusion and Attention mechanisms.	Kvasir-SEG	Dice: 0.8618
DDANet (Tomar et al., 2021)	Polyp	Dual-decoder architecture, feature residual blocks, and squeeze-and-excitation layers for channel-wise attention.	Kvasir-SEG	mIOU: 0.78
ACSNet (Zhang et al., 2020)	Polyp	ResNet34 encoder, with LCA and GCM modules with ASM to aggregate features *via* channel-wise attention.	Kvasir-SEG	mIOU: 0.838
PraNet (Fan et al., 2020)	Polyp	Res2Net encoder, parallel partial decoder, and reverse attention modules.	Kvasir-SEG	mIOU: 0.8295
CswinDoubleU-Net (Lin et al., 2024)	Polyp	U-shaped network composed of CNNs and Swin Transformer.	Kvasir-SEG	mIOU: 0.844
Focus U-Net (Yeung et al., 2021)	Polyp	Features a focus-gate to combine the spatial and channel attention in UNet-like architecture.	Kvasir-SEG	mIOU: 0.845
HarDNet-MSEG (Huang et al., 2021)	Polyp	HarDNet68 backbone and cascaded partial decoder	Kvasir-SEG	mIOU: 0.848
SMM-UNet (Li G. et al., 2025)	Tumor	Mamba UNet: Selective Fusion (SF) and Multi-scale Fusion (MF) Mamba modules.	BUSI	IOU: 0.688
FET-UNet (Zhang H. et al., 2025)	Tumor	Hybrid UNet: Parallel ResNet34 and Swin Transformer; AFAM module.	BUSI	IOU: 0.747
Anari et al. (2025)	Tumor	Multi-Branch Enc: ResNet + DenseNet + EfficientNet. Dec: CBAM and Non-local attention.	BUSI	IOU: 0.5305
Aumente-Maestro et al. (2025)	Tumor	Multi-task: Prediction-refining module; deterministic oversampling.	BUSI	DSC: 0.751
EMGA-Net Huang et al. (2025)	Tumor	Attention Fusion: Multi-scale group-mix (MGM) and Edge feature enhancement (EFE).	BUSI	mIOU: 0.8137
Zhang Y. et al. (2025)	Tumor	Enc-Dec: Multi-scale conv blocks (Enc) and Edge refinement layers (Dec).	BUSI	IOU: 0.7814
ARUNet (Liu H. et al., 2025)	Tumor	U-Net backbone enhanced with residual modules, attention mechanisms, and hybrid convolution.	BUSI	IOU: 0.7294
HEAT-Net (Jiang et al., 2023)	Tumor	U-Net-like model with CNN-transformer hybrid encoder, CdRBs, ECAT blocks, CDAM-enhanced skip connections, and a cascaded decoder.	BUSI	IOU: 0.767

## 3 Background

To effectively address the challenges of scale invariance and spatial resolution in dense prediction tasks, the proposed model utilizes several specialized techniques, and their fundamentals are discussed.

### 3.1 Global max pooling

**Figure 1:**
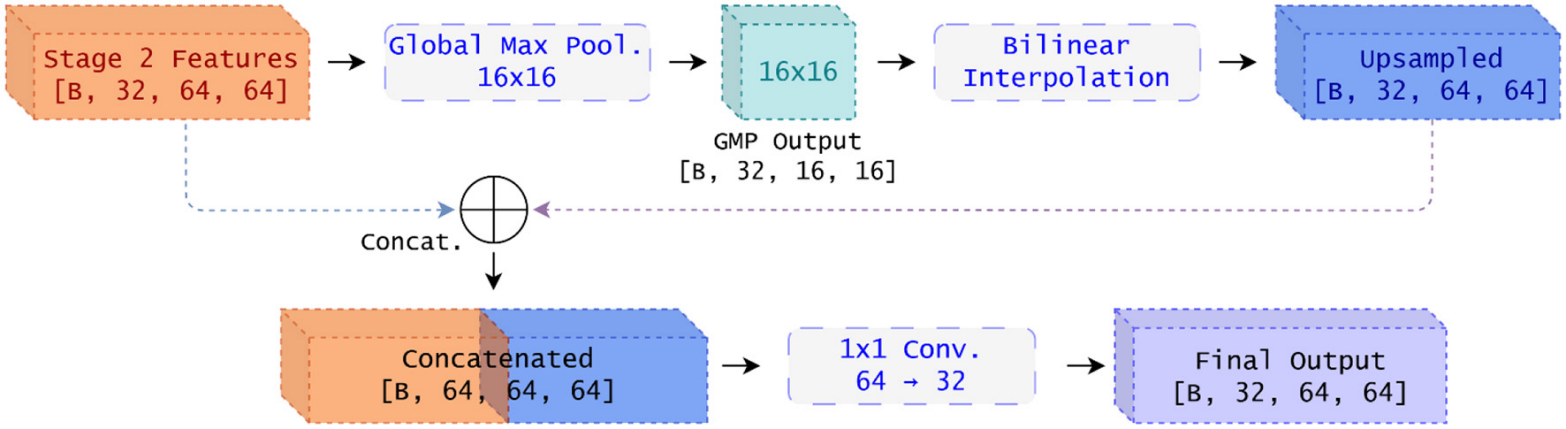
Block diagram representation of the global content aggregation block with global max. pooling with feature fusion.

The use of global max pooling enables explicit encoding of the dominant semantic activations, wherein the strongest response per channel is captured to extract the salient structures regardless of their spatial location. This reintroduction of the global semantic information into the spatial feature map restores the reinforcement of object-level awareness and aids in reducing the false positive rates introduced by the local texture ambiguity. The block diagram for the global max pooling operation is shown in Figure 1. The pooled vector can be expressed mathematically as in Equation 1:


$$\begin{array}{lll} {\hat{\text{F}}}_{\text{GMP}} & = & \text{Broadcast}\left(\underset{\text{i},\text{j}}{\max{\text{F}}^{\left(\text{s}\right)}\left(\text{c},\text{i},\text{j}\right)}\right) \end{array}$$ (1)

### 3.2 Spatial pyramid pooling for scale-aware regional context

**Figure 2:**
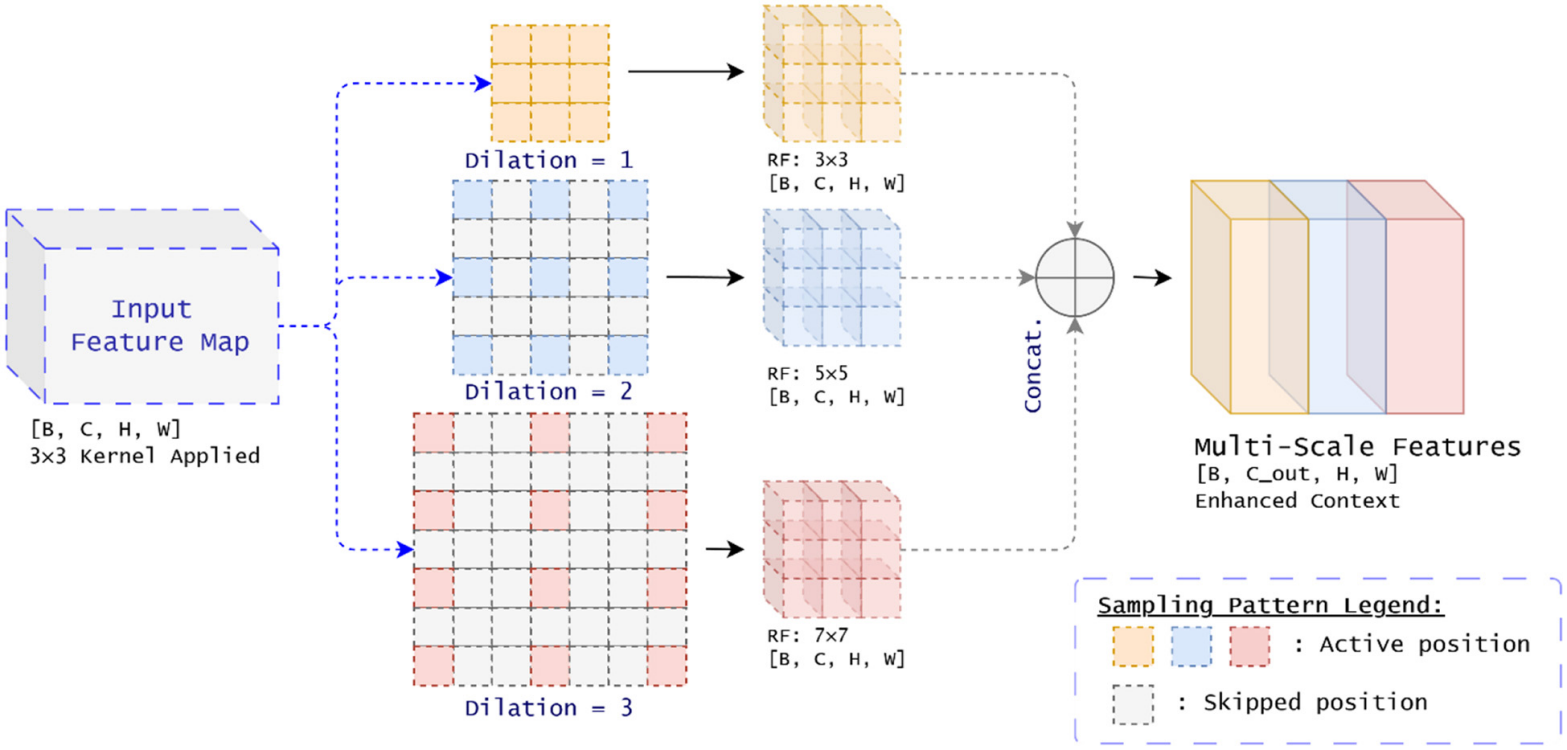
Block diagram representation of the dilated convolutions.

The medical segmentation problem characterizes significant scale variation, wherein the tumor size may vary in shape and size; to address it, the introduction of the spatial pyramid pooling enables aggregation of the contextual information at multiple spatial scales (as shown in Figure 2), which improves the robustness of the segmentation of objects of varying sizes and shapes by furnishing the encoder with coarse region-level context and fine spatial details. Mathematically, the feature map output is expressed as in Equation 2:


$${\text{F}}_{\text{SPP}}\text{ =}\overset{\text{K}}{\underset{\text{k=1}}{⊕}}U(P_{\text{k}}(F^{\left(s\right)}))$$ (2)

where, $P_{k}\left(\cdot \right)$ denotes pooling with bin size *k*, U(·) denotes upsampling, and ⊕ denotes the channel-wise concatenation.

### 3.3 Atrous spatial pyramid pooling (ASPP)

**Figure 3:**
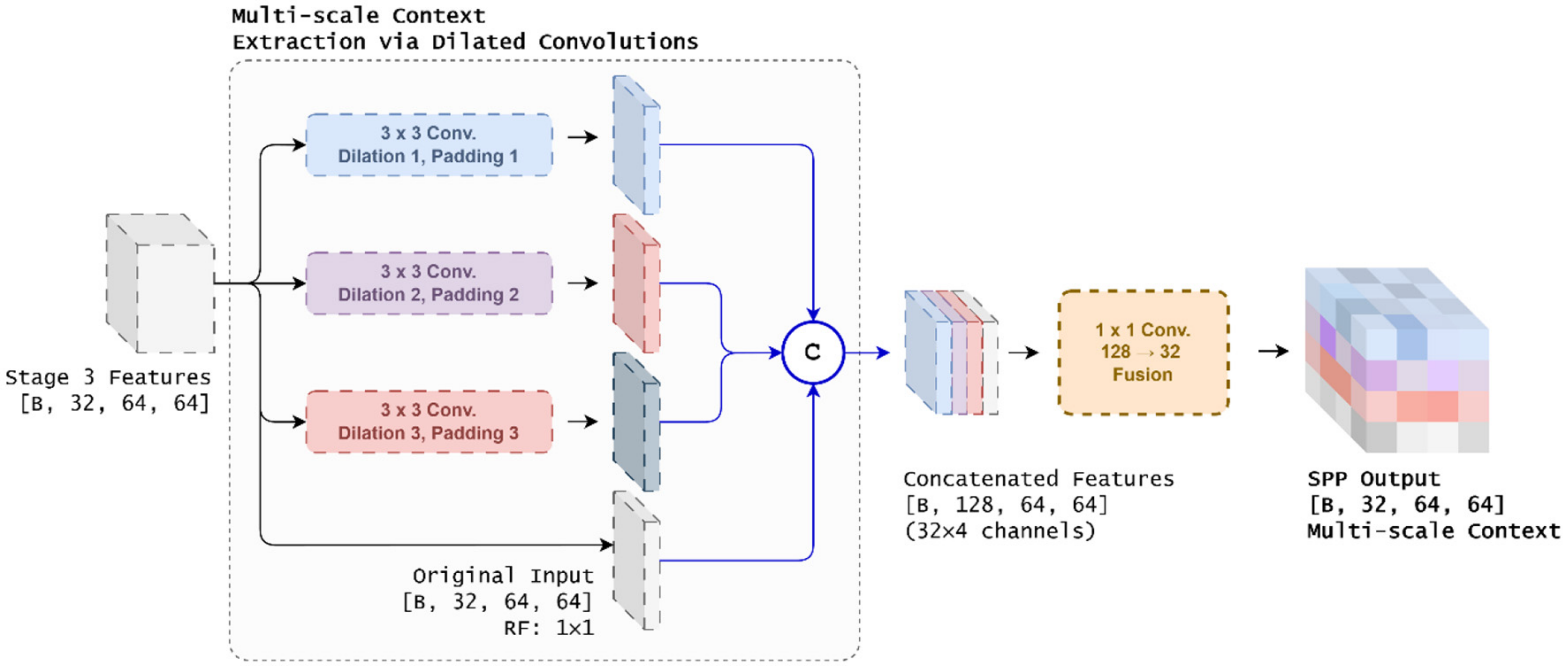
Spatial pyramid pooling with dilated convolutions.

**Figure 4:**
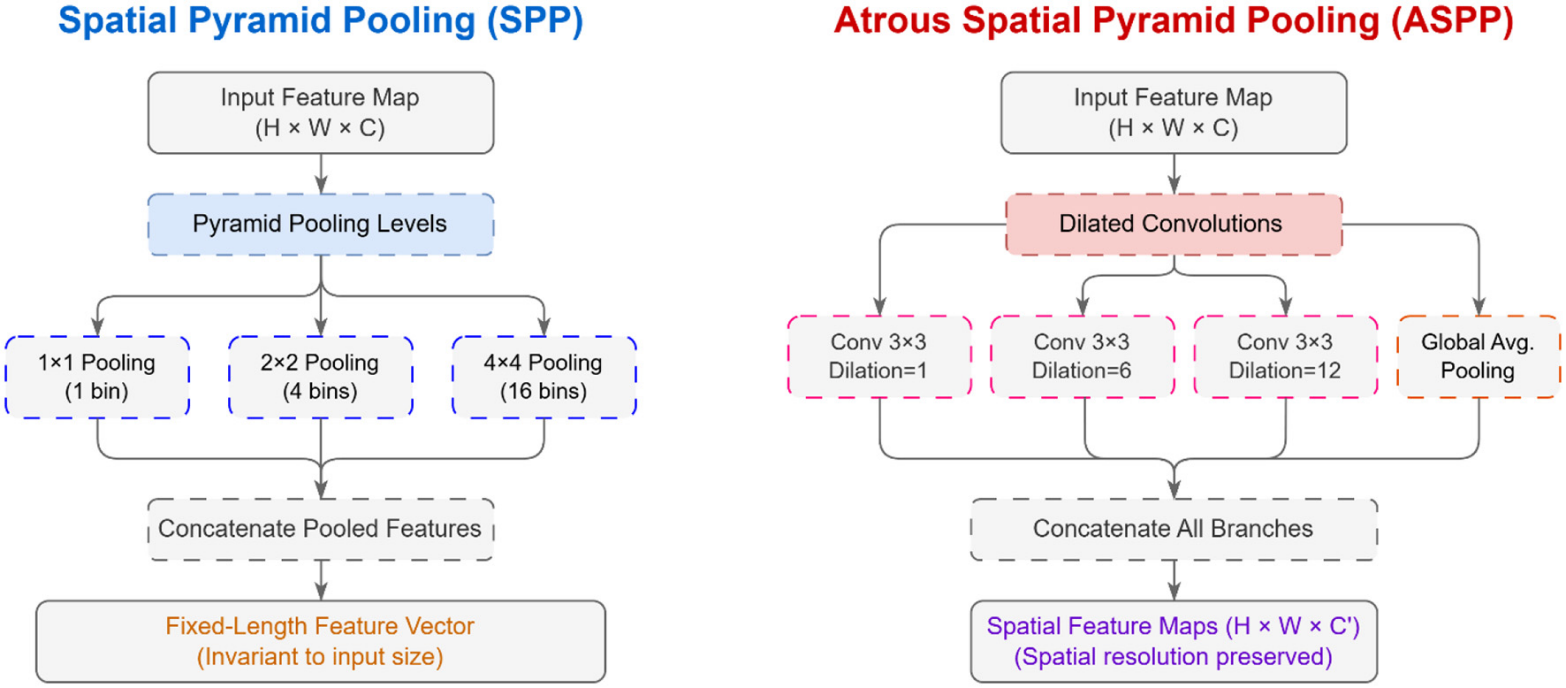
Comparison of the SPP and ASPP modules.

The ASPP module applies parallel Atrous convolutions to further expand the receptive field without degrading resolution, which can be visualized in Figure 3. Spatial pyramid pooling (SPP) and Atrous spatial pyramid pooling are two distinct multi-scale feature aggregation techniques. SPP produces fixed-length feature vectors using max pooling (1 × 1, 2 × 2, 4 × 4 bins) regardless of the size of the input leading to loss of spatial resolution due to aggressive downsampling; on the contrary, ASPP uses dilated convolutions at multiple learnable rates to expand the receptive field while preserving the spatial dimensions, while the receptive field in SPP is fixed. An architectural comparison between the two is presented Figure 4. Mathematically, it can be expressed as in Equation 3:


$$\begin{array}{lll} {\text{F}}_{\text{ASPP}} & = & \underset{r\in R}{⊕}\sigma \left(BN\left(W_{r}{*}_{r}F^{\left(s\right)}\right)\right) \end{array}$$ (3)

where $\overset{*}{r}$ denotes Atrous convolution with a dilation rate *r*, and the concatenated output is projected in Equation 4:


$${\text{F}}_{\text{ASPP}}^{'}\text{ = }W_{1\times 1}^{*}F_{ASPP}\in R^{256\times \frac{H}{4}\times \frac{W}{4}}$$ (4)

### 3.4 Spatial attention module (SAM)

**Figure 5:**
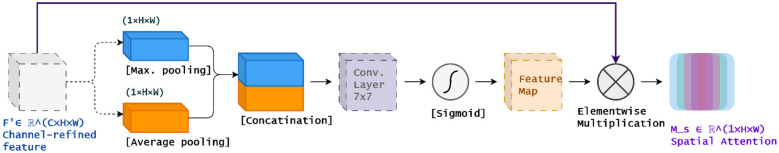
Block diagram representation of the spatial attention mechanism.

The spatial attention module, as shown in Figure 5, aids in enhancing the discriminative power of the network by exploiting the inter-spatial information by selectively emphasizing the key regions and suppressing irrelevant background noise. The SAM allows for addressing the artifacts prevalent in the shallow, high-resolution layers of the network; for example, these artifacts can be present in endoscopic images in the form of specular reflections, fluid debris, and motion blur. Let $F_{PRE}\in {\mathbb{R}}^{C_{pre}\times \frac{H}{2}\times \frac{H}{2}}$ represents the high-resolution features extracted by the pre-processing block, the SAM infers a two-dimensional spatial attention map *M*_*S*_ which highlights the most informative regions in the image (for example, polyp textures) while suppressing the irrelevant background information. The spatial attention map is computed by aggregating the channel information using both Average Pooling and Max Pooling operations along the channel axis; the 2D descriptors are represented as $F_{AVG}^{S}=AvgPool\left(F_{PRE}\right),\;F_{MAX}^{S}=MaxPool\left(F_{PRE}\right)$, and are further concatenated and processed by a convolution layer with a kernel size of 7 × 7 to capture effective local receptive fields, followed by the sigmoid activation function σ, represented mathematically in Equation 5:


$$\begin{array}{l} {\text{M}}_{\text{S}}\left({\text{F}}_{\text{PRE}}\right)=\text{σ}\left({\text{W}}_{\text{S}}^{*}\left[{\text{F}}_{\text{AVG}}^{\text{S}},\;{\text{ F}}_{\text{MAX}}^{\text{S}}\right]\right) \end{array}$$ (5)

where, $M_{S}\in {\left[0,1\right]}^{1\times \frac{H}{2}\times \frac{H}{2}}$; and the final refined feature map ${\tilde{F}}_{PRE}=F_{PRE}\;⊙\;M_{S}\left(F_{PRE}\right)$, where ⊙ represents the element-wise multiplication.

The lateral connection processing addressed the semantic gap of the deep features being semantically strong but spatially coarse; shallow features are spatially rich but noisy. The convolution blocks ensure that the channel depth of these features extracted at various depths is correlated. Along with that, the integration of the SAM also suppresses the artifacts, which are essential in medical image processing.

## 4 CA^2^PNet architecture

Semantic segmentation fundamentally relies on learning the feature representations that are simultaneously discriminative at the pixel level and contextually consistent at the region level, and leads to a principal conflicting challenge in segmentation of the requirements of larger receptive fields for semantic understanding and high spatial resolution for accurate boundary localization. The conventional segmentation models address this through aggressive spatial downsampling, which somehow improves the semantic abstraction but inevitably destroys fine spatial structure.

**Figure 6:**
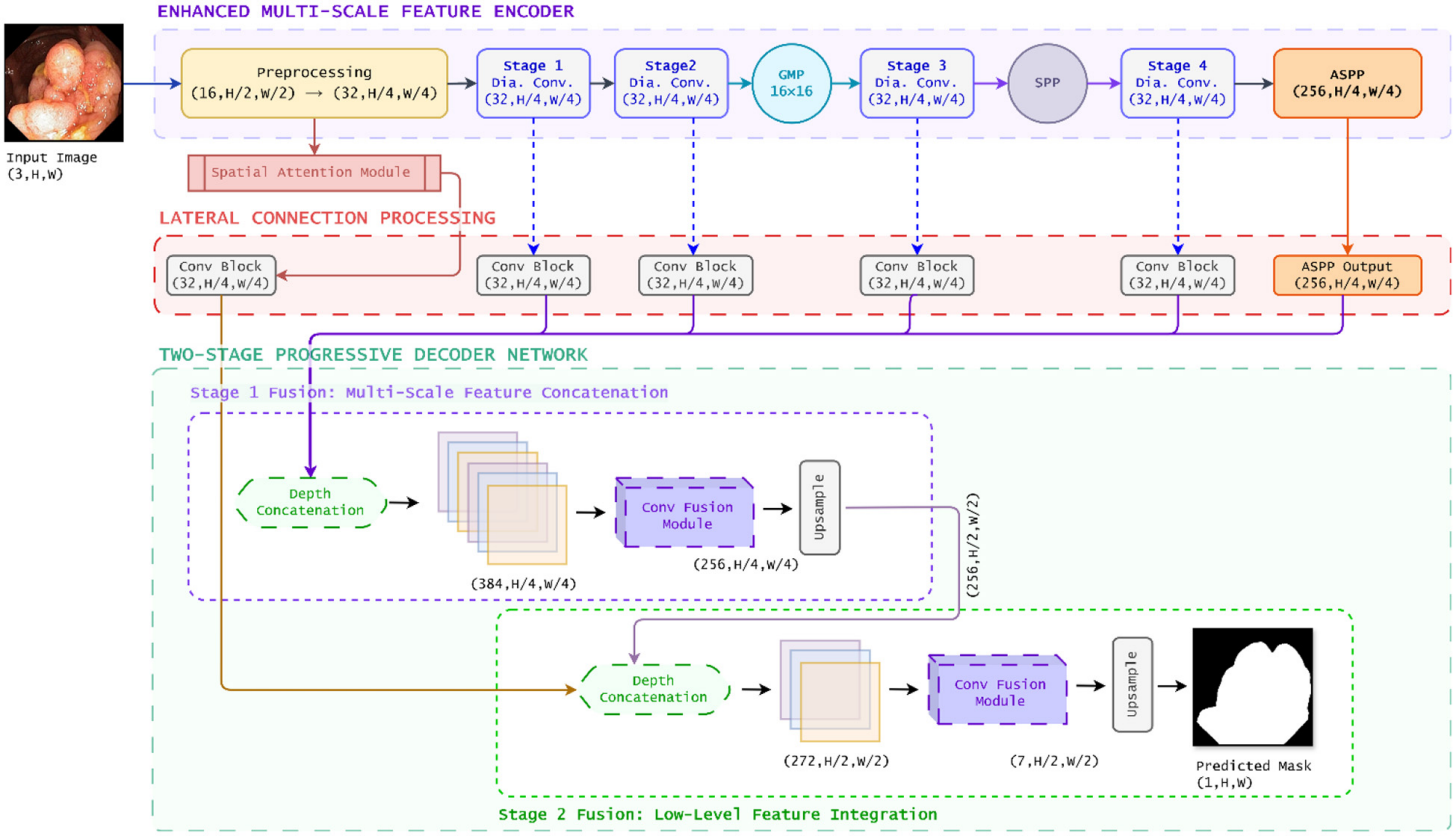
Architecture of the proposed CA^2^PNet architecture. The *Enhanced Multi-Scale Feature Encoder* (top, purple) extracts multi-scale contextual features using stage-wise convolutions, GMP, SPP, and ASPP modules. A lightweight *spatial attention module* refines encoder responses spatially (red). *The Two-Stage Progressive Decoder* (bottom, green) first concatenates deep multi-scale features and fuses them through a Conv. Fusion Module, producing a contextual map upsampled to Stage 1. Stage 2 concatenates the upsampled contextual features with low-level lateral features, performs detail refinement *via* another Conv Fusion Module, upsampled to full resolution, and outputs the predicted mask.

CA^2^PNet features a two-stage progressive segmentation network and draws inspiration from the DeepLabV3+ (Chen et al., 2018) and FusionNet (Liu C. et al., 2025). CA^2^PNet introduces an enhanced multi-scale encoder, spatial attention, lateral connections, and a two-step decoder that first fuses deep multi-scale context and then integrates low-level detail to enable precise segmentation. The model has been constituted to (a) capture expansive contextual features using multiple receptive fields (*via* the integration of stage-wise progressive dilated convolutions, global max. pooling, spatial pyramid pooling, and Atrous spatial pyramid pooling in the architecture at specific stages), (b) integrate an explicit spatial attention module for refining the spatially-important regions, and (c) achieve progressive fusion and upsampling for reconstructing high-definition segmentation masks. Figure 6 shows the detailed configuration of the CA^2^PNet model.

The overall configuration of the CA^2^PNet model follows an encoder–decoder architecture, wherein the encoder extracts the multi-scale features through progressive stages, with each stage incorporating dilated convolutions. The decoder features a two-stage fusion strategy to efficiently combine the features from different scales while preserving the spatial details to construct the segmentation masks. Mathematically, the model can be expressed as a mapping function F:X→Y, which transforms an input RGB image *X*∈ℝ^3 × *H*×*W*^ to a probabilistic segmentation mask Ŷ∈ℝ^1 × *H*×*W*^. The model has 47,77,940 trainable parameters.

The details of each of the modules are discussed below.

### 4.1 Enhanced Multi-Scale Feature Extractor (EMFE)

The proposed *Enhanced Multi-scale Feature Extractor (EMFE)* module is explicitly designed to address the shortcomings in conventional networks, wherein the proposed model relies on the multi-scale operations for achieving robust contextual modeling while preserving fixed spatial resolution for most encoder stages, rather than using conventional depth-induced receptive field expansion. Thus, enabling the network to capture local detail, global context, and scale-aware semantics within a unified representation. Let the input image be defined as **I**∈ℝ^3 × *H*×*W*^; the *EMFE* module generates an intermediate and final feature map of the form ${\text{F}}^{\left(s\right)}\in {\mathbb{R}}^{C\times \frac{H}{4}\times \frac{W}{4}},\;$where *s* = 1, …, 4 refers to the stages and *C* = 32 denotes the channel dimensionality of intermediate encoder stages. This feature embedding stage transforms the raw input image to a compact feature map using dilated convolution operations to achieve rich contextual modeling while preserving the fixed spatial resolution across most of the encoder stages. The downsampling is performed only once to obtain ${\text{F}}_{\text{0}}\in {\mathbb{R}}^{32\times \frac{H}{4}\times \frac{W}{4}}$, which will enforce the network to preserve the essential geometric and boundary-related information, allowing the deeper layers to operate on spatially meaningful representations rather than heavily compressed feature maps as in the case of conventional encoder–decoder-based architectures like UNet.

The EFME module [upper part (violet) of Figure 6] comprises four sequential encoder stages, *S*_1_ to *S*_4_, that apply parallel dilated convolution operation with varying dilation rates to extract multi-scale context simultaneously. This enables the encoder to sample features at varying spatial intervals simultaneously to encode the local, mid-level, and global features. Encoder features four sequential stages, each responsible for increasing the semantic abstraction while preserving spatial resolution ${\text{F}}^{\left(s\right)}=E^{\left(s\right)}\left({\text{F}}^{\left(s-1\right)}\right),s=1,\ldots ,4$; wherein each refinement block $E^{\left(s\right)}\left(\cdot \right)$ consisting of dilated convolution layers, followed by normalization and non-linearity, is defined as in Equation 6:


$$\begin{array}{lll} E^{\left(s\right)}\left(X\right) & = & \sigma \left(BN\left(W_{2}^{\left(s\right)}*\sigma \left(BN\left(W_{1}^{\left(s\right)}*X\right)\right)\right)\right) \end{array}$$ (6)

where, $W_{1}^{\left(s\right)}$ and $W_{2}^{\left(s\right)}$ denote convolution kernels applying stage-specific dilation, BN represents the batch normalization, and σ(·) is the sigmoid activation function.

These stacked dilated convolutional transformations progressively increase the receptive field across stages while maintaining full spatial correspondence. Unlike pooling-based encoders, semantic depth is achieved through repeated nonlinear transformations rather than resolution reduction, which is critical for maintaining boundary fidelity. The *EMFE* module also features three additional enhancements, wherein global max-pooling has been introduced after Stage-2 convolutions, followed by spatial pyramid pooling after Stage-3 convolutions and Atrous spatial pyramid pooling after Stage-4 convolutions to extract robust feature maps from each operation. The details of each are discussed as follows:

- *Global max. pooling* enables the explicit encoding of the dominant semantic activations, wherein the strongest response per channel is captured to extract the salient structures regardless of their spatial location.
- *Spatial pyramid pooling* after the Stage-3 convolutions enables aggregation of the contextual information at multiple spatial scales, which empowers the *EMFE* module to encode both the coarse region-level context and fine spatial details and aids in improving the robustness of the segmentation for objects of varying sizes and shapes. This allows the model to address the challenge of scale variation in medical segmentation problems.
- *Atrous spatial pyramid pooling* is applied after the Stage-4 convolutions to capture both the local texture information and long-range spatial dependencies and enable the encoding of the long-range dependencies, which is particularly essential for segmenting extended or disconnected structures.

These specific enhancements to the EMFE module enable accuracy of the segmentations by preserving spatial granularity by minimizing the downsampling, which in result preserves the boundary information within deeper features. The introduction of SPP and ASPP jointly handles the scale variability and enables the capture of long-range dependencies; the inclusion of global max-pooling reinforces the dominant object cues; and finally, the channel-wise concatenation maintains the separable contextual representations. Thus, together, these enhancements improve the extraction of finer feature embeddings which are semantically coherent, spatially precise, and scale-robust, directly leading to improved region consistency and boundary accuracy in segmentation. The final output, as in Equation 7, of the EMFE module integrates all the context-enhanced representations as given in the equation below, and this fusion strategy enables the preservation of the complementary information rather than prematurely converging it, allowingthe decoder to operate on a rich and expressive feature map.


$$F_{EMFE}=F^{\left(s\right)}⊕{\hat{F}}_{GMP}⊕F_{SPP}⊕F_{ASPP}^{'}$$ (7)

### 4.2 Lateral connection processing and attention

The architecture incorporates a dedicated lateral connection processing module for two critical tasks: (a) feature standardization and (b) saliency refinement, wherein the former ensures the channel consistency across multi-scale inputs and the latter employs the spatial attention to suppress the background noise in the low-level features. The feature maps with varying semantic depths generated *via* the EMFE module's encoder stages {*S*_1_, *S*_2_, *S*_3_, *S*_4_} maintain the spatial resolution and enable dense fusion strategy in the following decoding states. These heterogeneous features must be projected into a unified hyperspace. Let $F_{ENC}^{\left(i\right)}\in {\mathbb{R}}^{C_{i}\times \frac{H}{4}\times \frac{H}{4}}$ represents the feature map from the *i*^th^ encoder stage, where *i*∈{1, …, 4}, the lateral connection module applies the projection $\Phi_{LAT}^{\left(i\right)}$ to each stage given as in Equation 8:


$$\begin{array}{l} L_{i}=\Phi_{LAT}^{\left(i\right)}\left(F_{ENC}^{\left(i\right)}\right)\\ \;=ReLU\left(\mathrm{BN}\left(W_{LAT}^{\left(i\right)}*F_{LAT}^{\left(i\right)}+b_{LAT}^{\left(i\right)}\right)\right) \end{array}$$ (8)

where, ${\text{W}}_{LAT}^{\left(i\right)}$ is the learnable parameters of the 1 × 1 convolution kernel, and BN is the batch normalization.

The standardization ensures balancing the contribution of each scale when features are concatenated in the decoder, thus preventing high-dimensional stages from dominating the gradient flow.

Along with that, in this module, after the images are preprocessed, they are passed to the second decoder stage *via* a spatial attention module, as can be seen in the architecture diagram in Figure 6. The SAM has been integrated specifically to address the artifacts prevalent in the shallow, high-resolution layers of the network.

### 4.3 Two-stage progressive decoder network

The architecture features a two-stage progressive decoder network, wherein *Stage 1* enables semantic refinement by fusing the features from all encoder stages *S*_1_, …, *S*_4_ directly with the high-level ASPP features and *Stage 2* aggregates the semantic features from Stage 1 with attention-refined low-level features by integrating the SAM; this ensure that the final segments (after upsampling) are derived from the valid anatomical structure rather than artifacts.

#### 4.3.1 Stage 1: semantic aggregation stage

The first stage in the decoder simultaneously synthesizes the complete feature maps from all the encoding stages by concatenating them along the channel dimension; the high-level ASPP output is fused by the lateral outputs from all four stages *F*_*D*1_ = *F*_*ASPP*_⊕*F*_1_⊕*F*_2_⊕*F*_3_⊕*F*_4_. These aggregated features are processed by the convolution module $\left(F_{FUSE}\right)$ to reduce the channel dimensionality, followed by the bilinear upscaling $\left(U_{\times 2}\right)$ as in Equation 9.


$$\begin{array}{lll} D_{1} & = & U_{\times 2}\left(F_{FUSE}\left(F_{D1}\right)\right),\;D_{1}\in {\mathbb{R}}^{256\times \frac{H}{2}\times \frac{W}{2}} \end{array}$$ (9)

#### 4.3.2 Stage 2: low-level feature integration

The second stage of the decoder recovers the fine boundary by integrating the high-resolution features from the pre-processing block $F_{Pre}^{'}$. The features from the preprocessing block are processed *via* a spatial attention module to extract the finest spatial details like edges and textures but lack the semantic context. The input to the decoder's second stage is formed by concatenating the upsampled semantic features *D*_1_ from Stage 1 with these low-level features from the SAM $F_{D2}=D_{1}⊕F_{Pre}^{'},\;D_{2}\in {\mathbb{R}}^{272\times \frac{H}{2}\times \frac{W}{2}}$. These features *F*_*D*2_ undergo final processing *via* the second fusion module $F_{FUSE_{2}}$, which projects to seven channels followed by a final upsampling to original image resolution (*H, W*) and a pixel-wise prediction layer as given by Equation 10.


$$\begin{array}{lll} Ŷ & = & \sigma \left(U_{\times 2}\left({F_{FUSE}}_{2}\left(F_{D2}\right)\right)\right) \end{array}$$ (10)

where, σ is the sigmoid activation function.

These two-stage decoder stages enable late fusion, wherein the very high-resolution features $F_{Pre}^{'}$ are only introduced at the last stage, which avoids polluting the deep semantic feature extraction (Stage 1) with high-frequency noise. Stage 2 is purely responsible for boundary refinement, wherein the coarse segmented masks predicted by Stage 1 are snapped to the precise edges provided by the preprocessing block.

The architecture of the proposed model distinguishes itself from existing methods in the following key aspects:

- The majority of the existing works feature a classical encoder–decoder (Dumitru et al., 2023; Singh et al., 2025; Wang H. et al., 2025) and ensemble (Dumitru et al., 2023) architectures, which rely on traditional pooling mechanisms; wherein the feature maps are aggressively downsampled to a heavily compressed latent space (typically $\frac{H}{32},\;\frac{W}{32}$). In the proposed model, the EMFE module performs downsampling only once during the preprocessing stage and a high spatial ratio of $\frac{H}{4},\;\frac{W}{4}$ is maintained across all encoder stages. Thus the CA^2^PNet model is able to preserve high-frequency spatial details, which are essential for fine segmentation of the boundaries, which might get washed out due to aggressive boundaries as in Liu D. et al. (2025); Song and Shin (2024).
- Along with that, in Ige et al. (2023), multiple convolutional kernel sizes are used, and in Huang et al. (2025), Zhang Y. et al. (2025), multi-level feature attention is used, and these multi-scale operations are isolated; whereas in the proposed model, parallel dilated context blocks are intrinsically embedded at each stage of the encoder. This allows for addressing the scale variance continuously rather than at post-processing stage.
- Also, models like Liu D. et al. (2025) aim to enhance the feature representation at the decoder using enhanced skip connections using attention gates and squeeze-and-excitation, while Aumente-Maestro et al. (2025) use classical layer-by-layer symmetrical decoding for multi-task frameworks. The CA^2^PNet features two-stage progressive decoder architecture, wherein Stage 1 fuses all encoder depths simultaneously to construct a dense semantic feature vector; Stage 2 is exclusively reserved for late-stage boundary refinement *via* attention-guided pre-processing features. This aids in preventing the corruption of the deep semantic features at the decoder with high-frequency imaging noise from the shallow layers coming *via* the skip connections.
- Some of the recent works have incorporated vision transformers like FET-UNet (Zhang H. et al., 2025) and state-space models (Li G. et al., 2025; Zhang et al., 2024) to capture long-range dependencies and global context. In the present work, the global context mapping is achieved through cascaded parallel dilated convolutions rather than the self-attention matrices or state-space sequences.

## 5 Results

The proposed CA^2^PNet model is evaluated for two medical segmentation datasets; the details of the datasets used, the development environment setup, the segmentation result, and the performance comparison with the existing works are discussed in this section.

### 5.1 Datasets

#### 5.1.1 BUSI dataset

The model has been evaluated on the Breast Ultrasound Images (BUSI) dataset, a publicly available dataset collected by Al-Dhabyani et al. (2020) in 2018 from the Baheya Hospital in Cairo, Egypt. The dataset comprises of 780 ultrasound images obtained from 600 female patients (age between 25 and 75 years) acquired using a LOGIQ E9 ultrasound system. The images are categorized into three distinct classes of Normal (with 133 samples), Benign (with 437 samples), and Malignant (with 210 samples), along with the inclusion of the radiologist's annotated pixel-level ground truth masks for the malignant and the benign classes to support classification and segmentation tasks. The images in the dataset include variation in the breast density, tumor size, and speckle noise; this presents challenges in typical medical imaging modalities and makes it suitable for evaluating the robust deep learning architectures.

#### 5.1.2 Kvasir segmentation dataset

To further examine the performance of the model on distinct medical imaging modalities, the evaluation has been carried out for polyp segmentation, wherein the Kvasir-SEG dataset (Jha et al., 2020) is considered. Kvasir-SEG is a prominent open-access benchmark dataset for polyp segmentation curated by Jha et al. in 2020 from the larger Kvasir dataset. Kvasir-SEG features 1,000 polyp images captured using high-definition endoscopic imaging at Vestre Viken Health Trust in Norway. Kvasir-SEG features images with varying resolution from 332 × 487 to 1920 × 1072 pixels, presenting realistic clinical challenges like specular reflections, varying lighting conditions, and diverse polyp morphologies. Each image in the dataset is accompanied by the pixel-wise ground truth masks manually annotated by expert endoscopists and verified by experienced gastroenterologists. The summary of the dataset characteristics is highlighted in Table 2.

**Table 2:** Summary of dataset characteristics for BUSI and Kvasir-SEG.

Feature	BUSI dataset	Kvasir-SEG dataset
Domain	Breast ultrasound imaging	Gastrointestinal endoscopy
Modality	Ultrasound (B-mode)	Optical RGB (Colonoscopy)
Total images	780	1,000
Resolution	Varying (Avg. 500 × 500)	Varying (332 × 487 to 1920 × 1072)
Classes	3 (Normal, Benign, and Malignant)	2 (Polyp, Background)
File format	PNG	JPEG (Images), PNG (Masks)
Ground truth	Binary masks (Benign/Malignant)	Binary masks and bounding boxes
Source	Al-Dhabyani et al. (2020)	Jha et al. (2020)

### 5.2 Development setup

The model is developed using the PyTorch deep learning framework, and the training is implemented on a workstation with an Intel i7 processor, NVIDIA's 3060 12 GB GPU, and 64 GB RAM. The models are trained for 500 epochs with a batch size of 16, using the Adam optimizer with a learning rate of 1 × 10^−4^. To address the class imbalance between the lesion (foreground) and the background, a composite loss function, $L_{total}$ (as in Equation 11) which combines Binary Cross-Entropy $L_{BCE}$ (Equation 12) and Dice Loss $L_{Dice}$ (Equation 13) is considered. The binary cross entropy monitors the pixel-wise classification accuracy, whereas the dice loss ensures that the model focuses on the structural overlap of the segmented region, and is given mathematically as follows:


$$\begin{array}{lll} L_{total} & = & L_{BCE}+L_{Dice} \end{array}$$ (11)

$$\begin{array}{lll} L_{BCE} & = & -\frac{1}{N}\overset{N}{\underset{i=1}{\sum }}\left[y_{i}\cdot \log\left({ŷ}_{i}\right)+\left(1-y_{i}\right)\cdot \log\left(1-{ŷ}_{i}\right)\right] \end{array}$$ (12)

$$\begin{array}{lll} L_{Dice} & = & 1-\frac{2\sum_{i=1}^{N}y_{i}{ŷ}_{i}+\epsilon }{\sum_{i=1}^{N}y_{i}+\sum_{i=1}^{N}{ŷ}_{i}+\epsilon } \end{array}$$ (13)

Also, to enhance the model's generalization abilities and prevent overfitting, the data augmentations are applied with randomized flips (both horizontal and vertical) and intensity variations through brightness and contrast adjustments are also incorporated.

### 5.3 Segmentation results

#### 5.3.1 Segmentation on BUSI dataset

**Figure 7:**
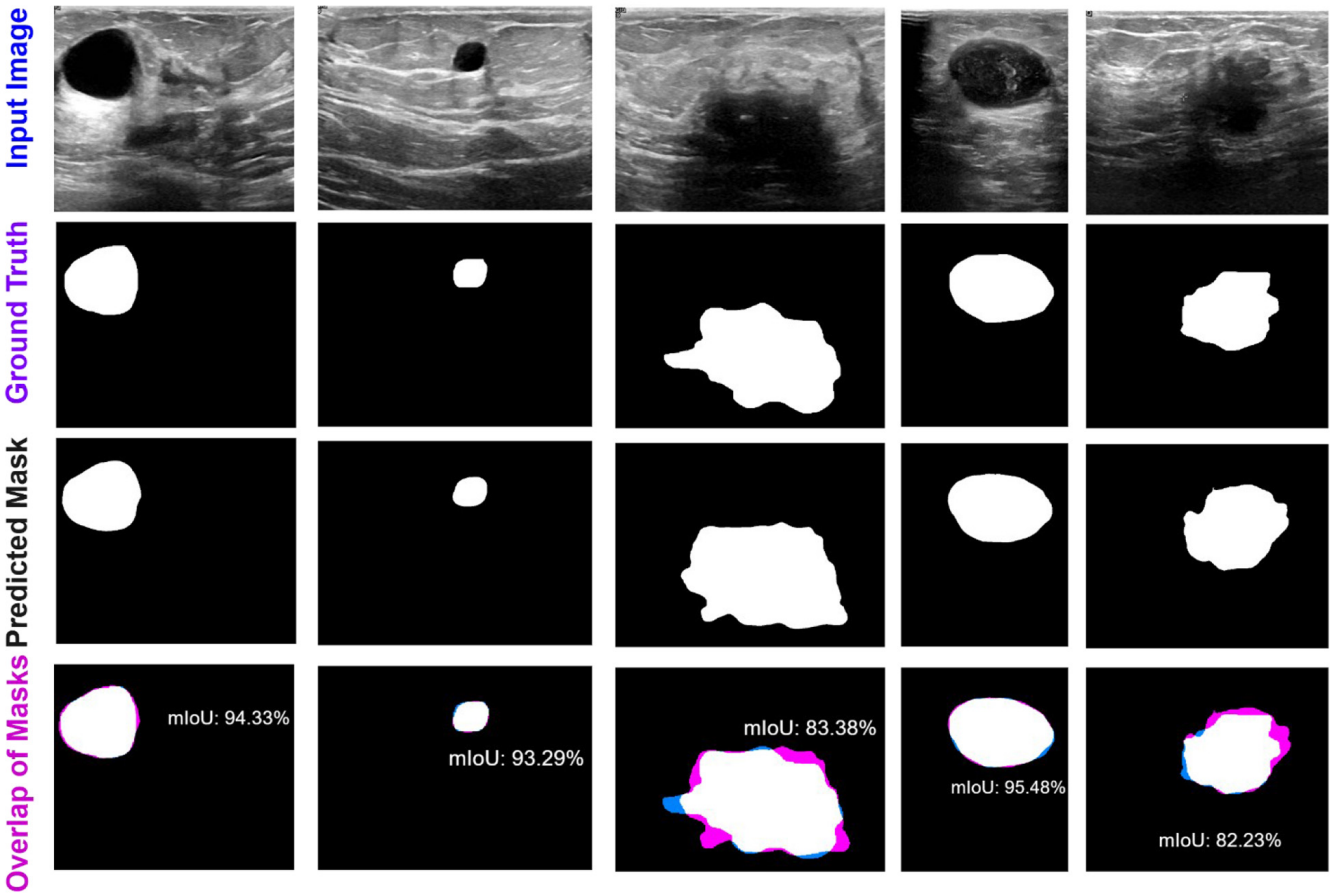
Segmentation results of the proposed model for the BUSI dataset, where white regions indicate True Positives (correct overlap), magenta regions highlight false positives (over-predicted areas), and blue regions denote false negatives (missed lesion areas).

To access the segmentation performance of the proposed model, the benign and malignant classes from the BUSI dataset are considered; the dataset is sampled uniformly at the image level, shuffled randomly each epoch, and iterated once per epoch without replacement to form batches, with augmentation applied only to training data. A total of 497 images were used for training the model and 150 images were kept for the test purpose. The various performance metrics for the held-out and the average values for the 5-fold cross-validation for the test set are listed in Table 3. The model achieves an F1-score of 89.99%, an accuracy of 96.55% on the test data, and a mean intersection over the union (mIOU) of 82.78%, which confirms the ability of the model to accurately demarcate the tumor boundaries, along with that a higher mean recall/sensitivity also minimizes the risk of false negatives. The segmentation results are shown in Figure 7. We have also validated the performance of the proposed model *via* 5–fold cross-validation, and the metrics are reported in Table 4. A slight reduction in the performance of the cross-validation results when compared to the hold-out results in Table 3 is observed. This performance drop can be attributed to the various experimental settings that have been adopted to reduce the computational cost and training time due to the hardware/GPU constraints. First, in the cross-validation results, to deal with the GPU/hardware constraints, the input image is resized to 352 × 352 from 512 × 512, which reduces the computational complexity but lowers the segmentation accuracy. Second, the early stopping is also incorporated with a patience of 50 epochs, as a result the training converged and stopped between 250 and 300 epochs instead of the initial 500 epochs; the inclusion of the early stopping aids in preventing overfitting and reduces training time, and it might lower the final performance. Despite these modifications, the model continues to offer strong and consistent segmentation performance across multiple folds.

**Table 3:** Performance summary of the CA^2^PNet model for test data for the BUSI dataset.

Metric (in %age)	mIOU	Mean precision	Mean recall	F1 score	Accuracy
Hold-out CV	82.78	89.33	90.67	89.99	96.55
5-fold cross validation	78.02	87.10	86.23	86.64	95.56

**Table 4:** Summary of 5-fold cross-validation metrics for test data for the BUSI dataset.

Fold	mIOU (%)	Precision (%)	Recall (%)	F1 Score (%)	Accuracy (%)
Fold 1	78.56	89.94	84.44	87.11	96.04
Fold 2	79.46	87.86	87.27	87.57	96.53
Fold 3	78.76	87.66	86.61	87.13	95.83
Fold 4	75.69	85.29	84.57	84.93	94.39
Fold 5	77.65	84.76	88.24	86.46	94.99

**Figure 8:**
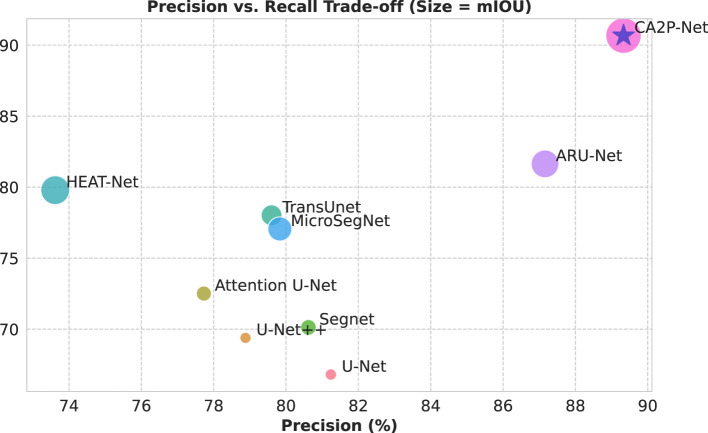
Precision-recall trade-off analysis for various segmentation models for the BUSI dataset (marker size is proportional to the mIOU).

The work has also been compared with the existing works already reported in literature in Table 5, and Figure 8 shows the precision-recall trade-off analysis for various segmentation models for the BUSI dataset, wherein each marker represents a specific architecture, with the marker size being proportional to the mIOU. The proposed CA^2^PNet (violet star) achieves the optimal balance between sensitivity and precision while maintaining the highest mIOU score.

**Table 5:** Compared performance indices for the BUSI dataset.

Method	mIOU (%)	Recall (%)	Precision (%)	F1 (%)	Accuracy (%)
U-Net (Liu H. et al., 2025)	60.00	66.81	81.24	69.32	95.43
U-Net++ (Liu H. et al., 2025)	59.93	69.39	78.88	69.75	95.30
Attention U-Net (Liu H. et al., 2025)	62.00	72.51	77.73	71.62	95.68
Segnet (Liu H. et al., 2025)	62.35	70.13	80.62	71.38	95.99
TransUNet (Liu H. et al., 2025)	66.20	78.02	79.60	75.53	96.14
HEAT-Net (Liu H. et al., 2025)	74.16	79.79	73.62	76.53	96.23
MicroSegNet (Liu H. et al., 2025)	69.12	77.06	79.83	78.42	96.55
ARU-Net (Liu H. et al., 2025)	72.94	81.64	87.16	81.86	96.74
*CA^2^PNet (Proposed)*	82.78	90.67	89.33	89.99	96.55

#### 5.3.2 Results from the Kvasir-SEG dataset

**Figure 9:**
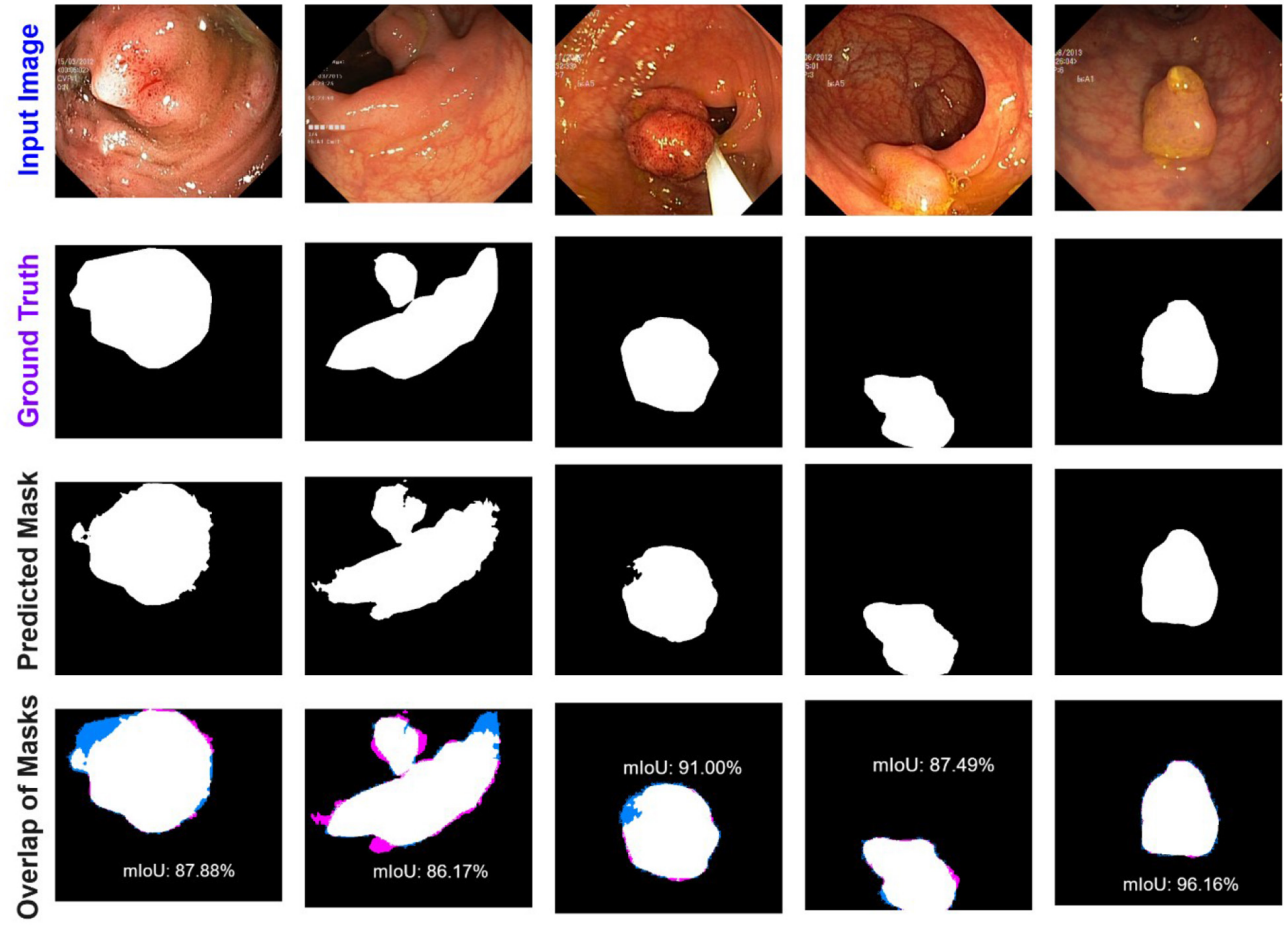
Segmentation results of the proposed model for the Kvasir dataset, where white regions indicate True Positives (correct overlap), magenta regions highlight False Positives (over-predicted areas), and blue regions denote False Negatives (missed lesion areas).

To further assess the model's performance, the evaluation is carried out on the Kvasir-SEG dataset. A total of 800 images were used for training the model, and 200 images were kept for the test purpose. The images are sampled uniformly and are shuffled randomly each epoch, iterated once per epoch without replacement to form batches, with augmentation applied only to training data. The various performance metrics for the test set are listed in Table 6. The model achieves an F1-score of 91.75%, an accuracy of 95.43% on the test data, and mean intersection over the union of 85.15%, which confirms the ability of the model to accurately demarcate the polyp's boundaries. The segmentation results are shown in Figure 9. We have also validated the performance of the proposed model *via* 5–fold cross-validation, and the metrics are reported in Table 7. A slight reduction in the performance of the cross-validation results when compared to the hold-out results in Table 6.

**Table 6:** Performance summary of the CA^2^PNet model for test data for the Kvasir-SEG dataset.

Metric (in %age)	mIOU	Mean precision	Mean recall	F1 score	Accuracy
Hold-out CV	85.15	93.13	90.41	91.75	95.43
5-fold cross validation	82.52	90.70	89.29	89.97	94.86

**Table 7:** Summary of 5-fold cross-validation metrics for test data for the Kvasir-SEG dataset.

Fold	mIOU (%)	Precision (%)	Recall (%)	F1 Score (%)	Accuracy (%)
Fold 1	84.87	91.44	91.57	91.50	95.52
Fold 2	83.45	90.48	90.69	90.59	95.15
Fold 3	86.78	93.43	91.95	92.69	96.31
Fold 4	82.43	89.44	90.33	89.88	95.18
Fold 5	75.09	88.75	81.94	85.21	92.15

**Figure 10:**
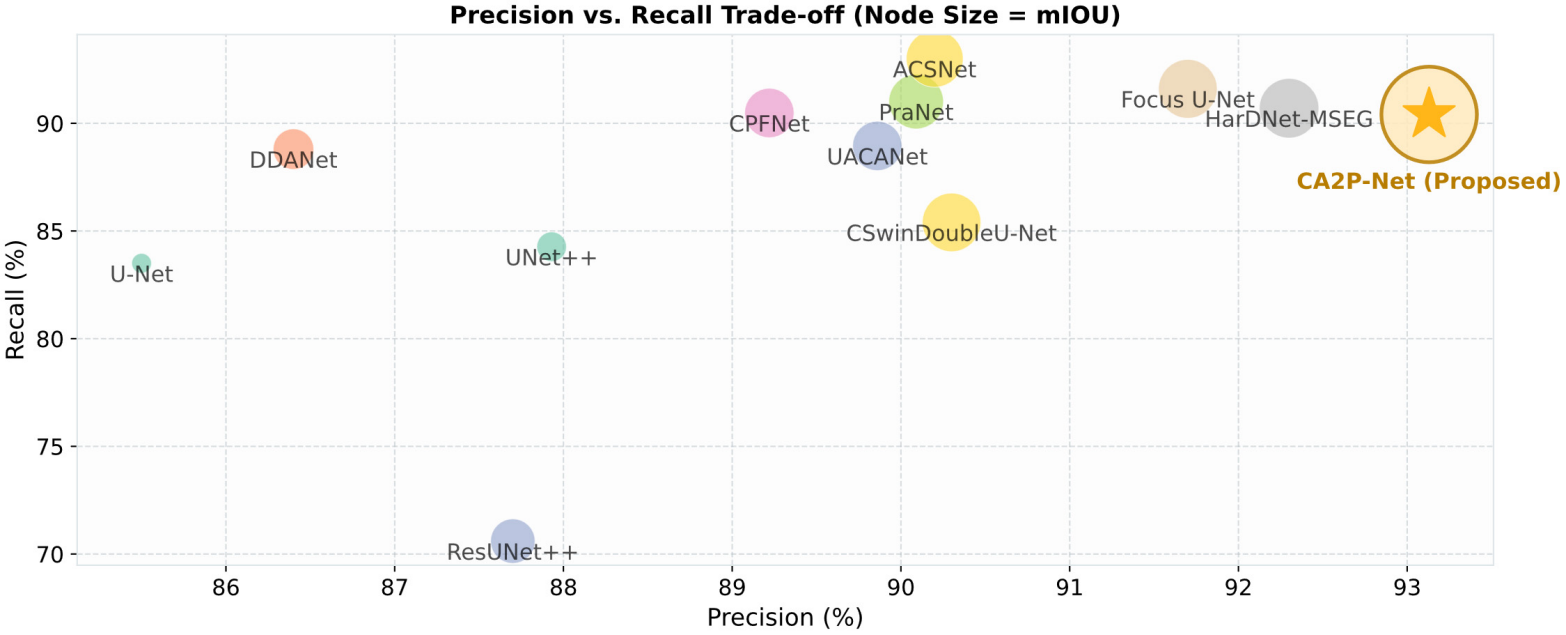
Precision-recall trade-off analysis for various segmentation models for the Kvasir-SEG dataset (marker size is proportional to the mIOU).

The work has also been compared with the works already reported in the literature in Table 8, and Figure 10 shows the precision-recall trade-off analysis for various segmentation models for the Kvasir-SEG dataset, wherein each marker represents a specific architecture, with the marker size proportional to the mIOU. The proposed CA^2^PNet (yellow star) achieves the optimal balance between sensitivity and precision while maintaining the highest mIOU score.

**Table 8:** Compared performance indices for the Kvasir dataset.

Methods	mIOU (%)	Recall (%)	Precision (%)
U-Net (Yu et al., 2026)	72.2	83.5	85.5
UNet++ (Wang K. et al., 2025)	74.71	84.27	87.93
DDANet (Tomar et al., 2021; Yu et al., 2026)	78.0	88.8	86.4
ResUNet++ (Yu et al., 2026)	79.3	70.6	87.7
UACANet (Wang K. et al., 2025)	80.99	88.95	89.86
CPFNet (Wang K. et al., 2025)	81.01	90.48	89.22
PraNet (Fan et al., 2020; Wang K. et al., 2025)	82.95	91.01	90.09
ACSNet (Yu et al., 2026; Zhang et al., 2020)	83.8	93.0	90.2
CSwinDoubleU-Net (Lin et al., 2024; Yu et al., 2026)	84.4	85.4	90.3
Focus U-Net (Yeung et al., 2021; Yu et al., 2026)	84.5	91.6	91.7
HarDNet-MSEG (Huang et al., 2021; Yu et al., 2026)	84.8	90.7	92.3
*CA^2^PNet (Proposed)*	85.15	90.41	93.13

## 6 Ablation studies

**Figure 11:**
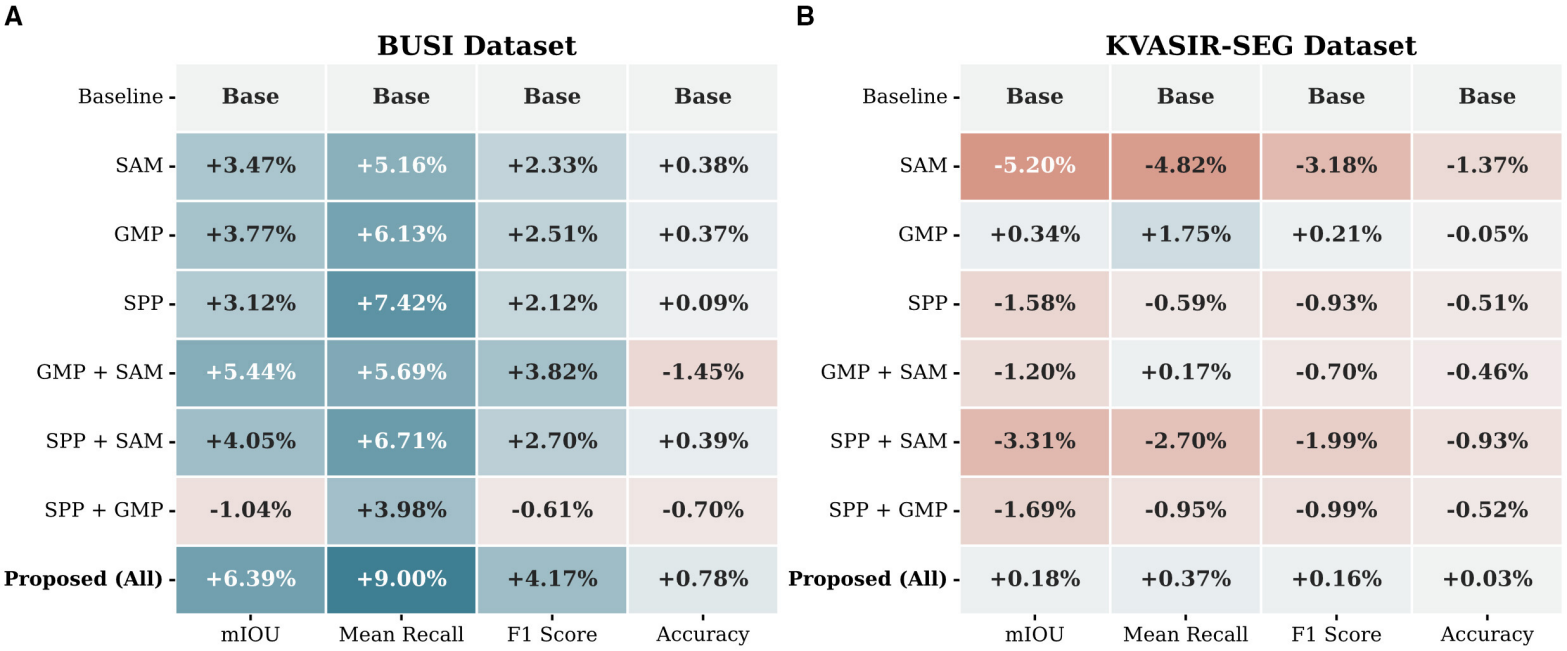
Heatmap illustrating the relative metric-wise performance improvement of each ablation configuration across the **(A)** BUSI and **(B)** Kvasir-SEG datasets.

The ablation studies have been included to evaluate the individual and joint contributions of the three components of SAM, GMP, and SPP; the ablation studies have been conducted for the initial configuration as reported in Section 5.2, and for both the datasets, the results have been reported in Table 9. For the base configuration (without SPP, GMP, and SAM) an mIOU of 77.81 and 85% and an F1 Score of 86.39 and 91.60% is achieved for BUSI and Kvasir-SEG datasets, respectively. Figure 11 shows the relative change in the performance metrics for the inclusion of the various modules in the architecture. In the case of the BUSI dataset, when compared to the baseline model, the complete configuration offers a relative improvement of 6.39% and a similar trend can be observed for mean recall wherein a relative improvement of 9% is observed. This highlights that the combination of the proposed modules ensures that the high-level semantic information is effectively aligned with the multi-scale spatial details. Also, in case of the Kvasir-SEG dataset, the baseline model already exhibits strong performance; however, it has also been observed that the isolated application of modules like SAM/SPP results in slight drop in the performance, which might be attributed to the misalignment of the features without global contextual balancing; however, the tripartite integration of the modules successfully recovers and stabilizes the network, eventually yielding a competitive mIOU of 85.15% and an accuracy of 95.43%. The ablation results also provide insights about the dataset-specific characteristics, wherein, in the case of high noise modalities like ultrasound, significant improvements are observed, thus proving the efficacy of the model on complex and ambiguous ultrasound imagery. Thus, the concurrent use of SPP, GMP, and SAM prevents the feature suppression and is critical for achieving semantic visualization in specialized, highly variable medical imaging modalities.

**Table 9:** Ablation studies for analyzing the impact of the various components in the CA^2^PNet model.

*SPP*	*GMP*	*SAM*	mIOU	Mean precision	Mean recall	F1 score	Accuracy
BUSI dataset							
**-**	**-**	**-**	77.81	90.53	83.18	86.39	95.80
**-**	**-**	✓	80.51	89.38	87.47	88.40	96.16
**-**	✓	✓	82.04	91.78	87.91	89.69	94.41
✓	**-**	✓	80.96	88.68	88.76	88.72	96.17
-	✓	-	80.74	88.85	88.28	88.56	96.15
✓	-	-	80.24	87.18	89.35	88.22	95.89
✓	✓	-	77.00	85.26	86.49	85.86	95.13
✓	✓	✓	**82.78**	**89.33**	**90.67**	**89.99**	**96.55**
*Kvasir—SEG dataset*							
**-**	**-**	**-**	85.00	93.32	90.08	91.60	95.40
**-**	**-**	✓	80.58	92.63	85.74	88.69	94.09
**-**	✓	✓	83.98	91.74	90.23	90.96	94.96
✓	**-**	✓	82.19	92.37	87.65	89.78	94.51
-	✓	-	85.29	91.92	91.66	91.79	95.35
✓	-	-	83.66	92.09	89.55	90.75	94.91
✓	✓	-	83.56	92.35	89.22	90.69	94.90
✓	✓	✓	**85.15**	**93.13**	**90.41**	**91.75**	**95.43**

Bold values indicate best results.

**Figure 12:**
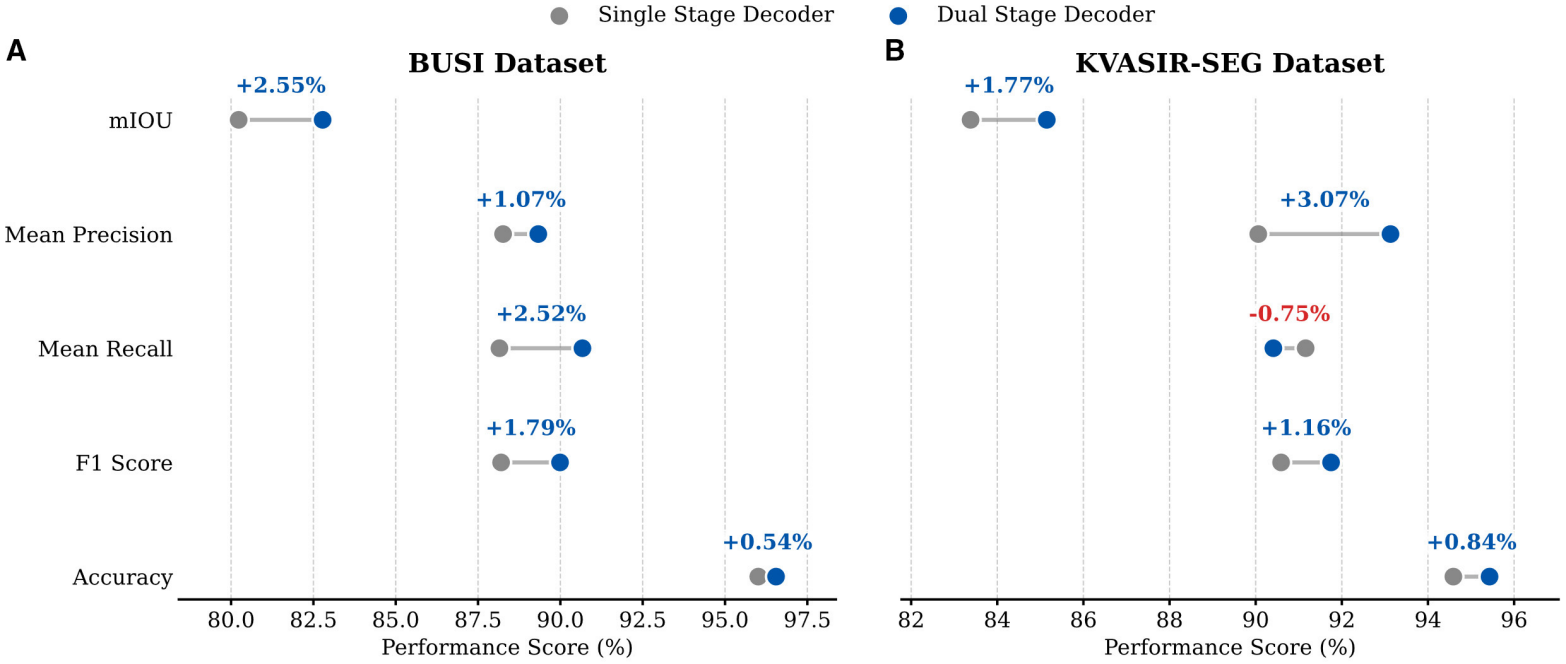
Dumbbell plot illustrating the absolute metric-wise performance improvement in single- and dual-stage decoder configuration across the **(A)** BUSI and **(B)** Kvasir-SEG datasets.

Table 10 presents the ablation studies for evaluating the efficacy of the proposed dual-stage decoder stage, wherein the Stage 2 decoder block, as seen in Figure 6, is removed, and the multi-scale encoder features are used by the Stage 1 decoder to perform the segmentation for both the datasets. The obtained results highlight that the use of a dual-stage decoding mechanism offers consistent and notable performance gains. For the BUSI dataset, the mIOU and F1-score have improved by 2.55 and 1.79% respectively; the model also offers better feature localization and segmentation, which can be attributed to an increase of mean recall by 2.52%. Similarly, a positive improvement is also observed in the case of the Kvasir-SEG dataset, wherein the dual-stage decoder improves the mIOU by 1.77% (absolute gain), along with the positive increase in the F1 Score and accuracy metrics. The same improvements have been visualized using dumbbell plots, as shown in Figure 12.

**Table 10:** Ablation studies for the single and dual stages in the decoder network.

Dataset	Decoder stages	mIOU	Mean precision	Mean recall	F1 score	Accuracy
BUSI	Single	80.23	88.26	88.15	88.20	96.01
	Dual	**82.78**	**89.33**	**90.67**	**89.99**	**96.55**
Kvasir-SEG	Single	83.38	90.06	91.16	90.59	94.59
	Dual	**85.15**	**93.13**	**90.41**	**91.75**	**95.43**

Bold values indicate best results.

## 7 Statistical analysis

**Figure 13:**
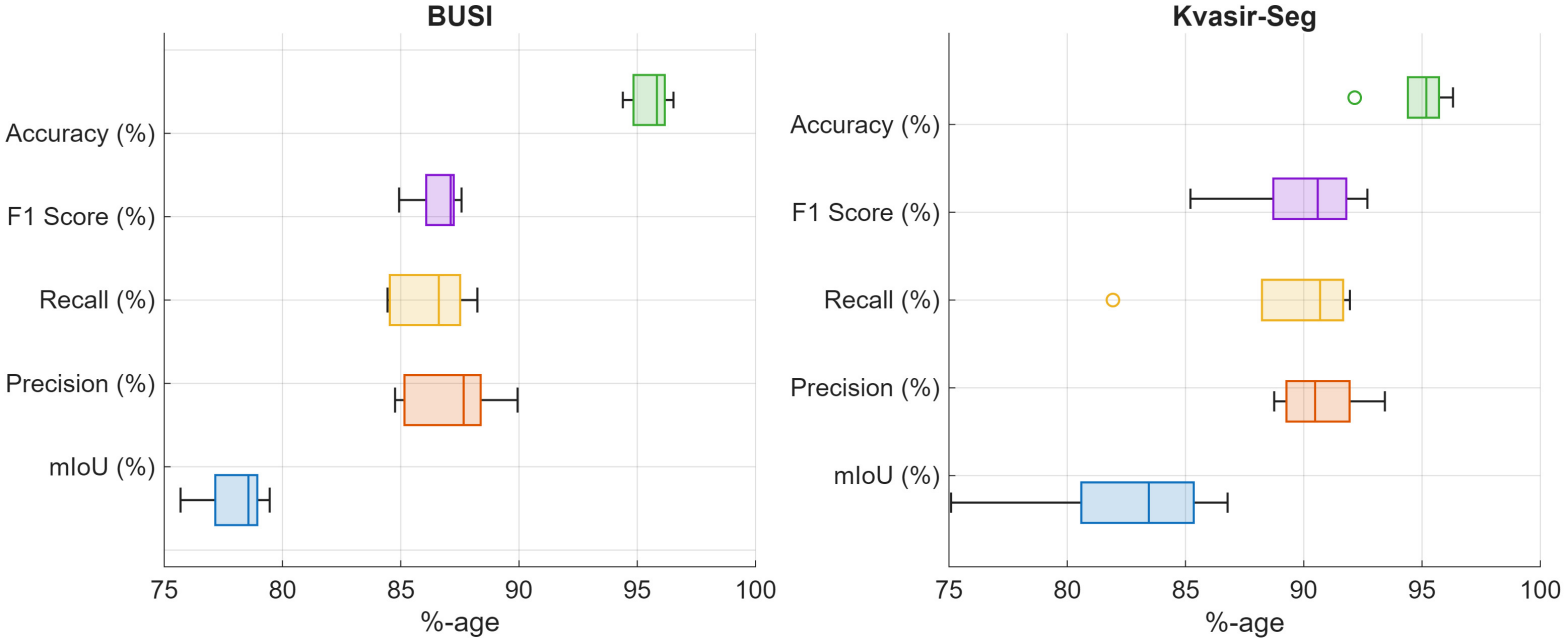
Box plot for the performance metrics of 5-fold cross-validation.

To validate the robustness of the model, statistical analysis is incorporated for the 5-fold cross-validation results. The mean and the standard deviation of the performance metrics for the 5-fold cross-validation results are given in Table 11, and the box plot for the same is shown in Figure 13. To further evaluate the generalization abilities of the proposed model across distinct imaging modalities, a comparative statistical significance is assessed using both the non-parametric Mann-Whitney U test and a Paired *T*-Test on the 5-fold cross-validation results obtained for the BUSI (ultrasound) and Kvasir-Seg (endoscopy) datasets. The Mann-Whitney U test offers a robust evaluation of the overall performance distributions without assuming data normality, and the Paired *T*-Test evaluates mean differences between paired observations when normality is assumed. The significance values for the various tests are given in Table 12.

**Table 11:** Mean and standard deviation for the performance of 5-fold cross-validation.

Metric	BUSI (Mean ±SD)	Kvasir-Seg (Mean ±SD)
mIOU	78.02 ± 1.46	82.52 ± 4.47
Precision	87.10 ± 2.10	90.71 ± 1.83
Recall	86.23 ± 1.68	89.30 ± 4.16
F1 score	86.64 ± 1.03	89.97 ± 2.86
Accuracy	95.56 ± 0.86	94.86 ± 1.59

**Table 12:** Statistical analysis for the performance of 5-fold cross-validation.

Metric	Mann-Whitney U test (*p*-value)	Paired *T*-test (*p*-value)	Significance (*p* < 0.05)
mIOU	0.1508	0.0750	Not Significant
Recall	0.1508	0.2728	Not Significant
F1 score	0.0952	0.0524	Not Significant
Accuracy	0.6905	0.3517	Not Significant

The clinical viability of the proposed segmentation model in detecting the lesions regardless of the imaging modality is evaluated by the statistical analysis of recall, and it can be observed in Table 12 that the model offers stability. None of the tests, the independent Mann-Whitney U test (*p* = 0.1508) and the controlled Paired *T*-Test (*p* = 0.2728), found any statistically significant difference in recall amongst BUSI and Kvasir-Seg segmentation. Similarly, for the mIOU (*p* = 0.1508 and *p* = 0.0750) and accuracy (*p* = 0.6905 and *p* = 0.3517) for both tests, no significant variance is observed. Thus, the lack of strict statistical significance across the metrics confirms that the model is equally proficient in segmenting the target regions across both modalities.

## 8 Discussions and limitations

The proposed model has the potential to assist clinical decision making; to validate the architecture's versatility, the evaluation has been carried out across two fundamentally distinct biomedical imaging modalities, viz. optical colonoscopy and breast ultrasound. The model relies on the *EMFE* module for the feature extraction; it was able to identify intra-class morphological variance of polyps and, in the case of ultrasound modality, which features speckle noise and low contrast boundaries. The use of a two-stage progressive decoder aids in delineating the tumors, wherein the decoder aggregates the global semantic context prior to the boundary delineation and has been successful in segmenting the malignant cases without being corrupted by the acoustic noise. Along with that, the ablation studies presented in Table 9 reveal a complex relationship between the inclusion of the SPP, SAM, and GMP in the encoder stage, which are inherited due to the complex characteristics of medical imaging modalities. In the case of the Kvasir-SEG dataset, the baseline model achieves a higher mIOU score of 85% and can be attributed to the endoscopic imagery; thus, the inclusion of modules like SPP and SAM initially leads to performance degradation and can be attributed to feature over-fitting. In the case of high-contrast environments, the inclusion of SPP leads to the amplification of the benign mucosal textures or light reflections; without the global context, the SAM may rigidly align these noise-induced artifacts with the decoder features, resulting in false-positive segmentations. However, the inclusion of GMP contextualizes local features and suppresses the ones that do not align with the global semantic profile of the lesion. Thus, it has been observed in the results that for the Kvasir-SEG, the full model offers an mIOU of 85.15%, wherein the GMP stabilizes the precision of the SPP and SAM. Conversely, in the case of the BUSI dataset, the baseline model offers a lower mIOU of 77.81%, which is characterized by speckle noise and poor acoustic contrast, thus making the baseline model underinformed and resulting in poor boundary delineation. Thus, the inclusion of modules like SPP, SAM, and GMP offers necessary complementary information for the localization of the tumor, and thus, offers a maximum mIOU of 82.78%. The proposed model shows successful application across diverse datasets and demonstrates that the multi-scale feature integration and dual-stage decoder for boundary refinement aids in yielding a robust, generalizable architecture.

However, despite the proposed advancements, the integration of the parallel multi-scale branches adds to the model's computational complexity and memory requirements and might pose challenges for deployment on edge devices. Along with that, the current framework processes the data on individual images, but in the case of live endoscopic video feeds, which might experience occasional inter-frame flickering during rapid camera movements, the temporal continuity is essential. The future work will aim to focus on integrating temporal attention for video consistently, extending the model to handle three-dimensional volumetric data, and further methodologies that can be employed to make the model lightweight for edge deployment.

## 9 Conclusions

The present work proposes a Context Aware Adaptive Progressive Network (CA^2^PNet) for the precise segmentation in medical images; it tries to address the challenges associated with the biomedical images because of anatomical complexity, inter-patient diversity, class imbalance, and irregular morphological patterns, using a series of modifications, like the integration of SAM to emphasize discriminative spatial regions, GMP to strengthen contextual representation and suppress background noise, and SPP for robust multi-scale feature extraction. The architecture allows for the simultaneous refinement of the extraction of local features as well as preserving the global context. The encoder features an Enhanced Multi-Scale Feature Extractor, which integrates parallel multi-scale context blocks at each state and enables a fixed spatial resolution throughout the encoding process; the decoder features a two-stage procedure, wherein the first stage fuses all the features from all encoder stages to locate the lesion and the second stage integrates the attention-refined high-resolution features *via* SAM to precisely recover the object boundaries. The work has been evaluated for the segmentation of the lesions in ultrasound images for breast cancer diagnosis and also for the segmentation of the gastrointestinal polyps using endoscopic images. Mean intersection over union values of 0.8278 and 0.8515 are achieved on evaluation for the BUSI and the Kvasir-SEG datasets, respectively. Statistical validation using a non-parametric Mann-Whitney U test and a Paired *T*-Test on the 5-fold cross-validation results has also been incorporated to validate the robustness of the model.

## Data Availability

Publicly available datasets were analyzed in this study. The dataset used in this study is available at: BUSI Dataset (Zhang et al., 2020): https://scholar.cu.edu.eg/?q=afahmy/pages/dataset is available the following link https://scholar.cu.edu.eg/Dataset_BUSI.zip and Kvasir-SEG (Fan et al., 2020): https://datasets.simula.no/kvasir-seg/.
